# Child and school staff perceptions and experiences of universal social and emotional learning curricula in context: A qualitative case study registered report examining ‘Passport Skills for Life’

**DOI:** 10.1111/bjep.70065

**Published:** 2026-03-02

**Authors:** Ola Demkowicz, Annie O'Brien, Suzanne Hamilton, Lauren Burke, Melek Alemdar, Carla Mason, Molly Anderton, Pratyasha Nanda, Lily Corke Butters, Felicitas Beissel, Yizhuo Lu, Eleanor Chatburn, Neil Humphrey, Pamela Qualter

**Affiliations:** ^1^ Manchester Institute of Education The University of Manchester Manchester UK; ^2^ Common Room North Leeds UK; ^3^ Department of Clinical Psychology and Psychological Therapies, Norwich Medical School University of East Anglia Norwich UK

**Keywords:** implementation, school‐based intervention, social and emotional learning, universal intervention

## Abstract

**Background:**

There is increasing interest in the circumstances under which universal school‐based social and emotional learning (SEL) interventions can be most effective, and how implementation moderates intervention outcomes. We focus on the implementation of ‘Passport Skills for Life’, an SEL intervention that has been introduced into over 100 schools in England to date.

**Aim:**

We examined the experiences and perceptions of both school staff and children in delivering and engaging with Passport.

**Method:**

We report on the implementation and process evaluation of a large cluster randomized controlled trial conducted in mainstream primary schools across Greater Manchester and surrounding areas. We used a qualitative longitudinal design, engaging with children, teachers and senior leaders in five case study schools via focus groups and interviews. We used reflexive thematic analysis to build an integrated picture, guided by relevant implementation theory on dimensions of, and factors affecting, implementation. We triangulated and examined differences in thematic representation among participant groups to understand differences in perspectives.

**Findings:**

Teachers generally *reported* high fidelity, but commonly made adaptations. The comic book format, emotional reflection opportunities and sensitive content of Passport were considered distinctive and valuable, though implementation was shaped by factors including school context and limited programme differentiation for children with SEND. Children, teachers and SLT were generally aligned in perspectives, with greater *within*‐group divergence.

**Conclusions:**

Findings highlight the need for inclusive, sensitive and context‐responsive SEL design, alongside guidance and support to ensure fidelity without compromising accessibility, impact or flexibility.

## INTRODUCTION

Social and emotional skills promote current and future mental health and well‐being among children and adolescents (Goodman et al., 2015). In England, the Partnership for Children has introduced ‘Passport Skills for Life’^1^ (hereafter ‘Passport’) (Partnership for Children, 2019), a social and emotional learning (SEL) intervention, into over 100 schools so far, highlighting its theoretical premise and emerging evidence base (Mishara & Dufour, 2020). However, there is no independent efficacy evidence, and in our randomized controlled trial (RCT), we have been determining whether Passport could reduce internalizing symptoms (primary outcome) and related outcomes compared to usual practice (control). This trial included an implementation and process evaluation examining the experiences and perceptions of school staff and children in delivering and engaging with the intervention. Qualitative methods have been generally underutilized in this area, particularly across different groups involved in the process (Durlak & DuPre, 2008; Rojas‐Andrade & Bahamondes, 2019), yet offer critical insight into dynamic processes within implementation and are crucial to understanding programme success or failure (Greenberg et al., 2005; Nilsen, 2022).

### Social and emotional learning

Emotional competence refers to two broad skills, namely the ability to (1) understand, express and regulate one's own emotions and (2) understand others' emotions. Social competence is the ability to interact with others effectively and in a prosocial manner (Bergin et al., 2023). Social and emotional learning (SEL) refers to the process through which children develop social and emotional competence: through SEL, children and youth acquire and learn to effectively apply the knowledge, attitudes and skills necessary to understand and manage emotions, set and achieve positive goals, feel and show empathy, establish and maintain positive relationships and make responsible decisions (Weissberg et al., 2015).

Schools are well placed for teaching SEL because they can provide learning environments that are safe, caring, well managed and participatory (Humphrey, 2023), and they have wide reach, a prolonged period of engagement and a central role in communities (Greenberg & Abenavoli, 2017). Universal school‐based interventions target all children regardless of level of difficulties or risk, which makes them non‐stigmatizing; they also have the potential to prevent or reduce problems predicted by shared or common risk factors, or disorders that will develop later in life, but are not manifested in childhood (Greenberg & Abenavoli, 2017).

Meta‐analytic evidence indicates that participation in universal school‐based SEL interventions results in significantly improved social and emotional skills, attitudes, behaviours, school climate and safety, peer relationships, school functioning and academic achievement (Cipriano et al., 2023); and decreases conduct problems and emotional distress (Durlak et al., 2022). Possessing good social and emotional skills protects against developing later mental health difficulties (Panayiotou et al., 2019; Sklad et al., 2012; Taylor et al., 2017) and is associated with higher levels of life satisfaction and wellbeing up to 18 years later (Goodman et al., 2015; Taylor et al., 2017).

There is increasing focus on the circumstances under which universal school‐based SEL interventions can be most effective, and we do not yet fully understand how or why implementation moderates intervention outcomes (Cipriano et al., 2023; Durlak et al., 2011).

### Passport Skills for Life

Passport (Partnership for Children, 2019) is a curriculum‐based SEL intervention for children aged 9–11 years. Passport aims to empower children to develop effective coping strategies and enable them to navigate challenges in the primary‐secondary school transition (Partnership for Children, 2019). It helps children develop coping mechanisms including emotion regulation, self‐management and responsible decision‐making so they can successfully deal with problems in their daily lives (Mishara & Dufour, 2020). Passport also aims to improve children's social skills, so that they can accurately identify and communicate their feelings, ask for help and support others (CASEL, 2023; Robertson et al., 2022).

Designed to be delivered by trained classroom teachers in five modules/18‐week sessions (see Table S1), Passport uses a comic format with engaging activities including stories, discussions, role‐play and games. Intervention materials include curricula, posters, comic strips, personalized ‘passports’, emotion cards, certificates of participation and home activities with parents (Partnership for Children, 2019). Teachers play a guiding role, fostering a supportive and inclusive atmosphere where children learn to ask for help when needed and to be willing to help others.

Positive effects of utilizing Passport as intended were demonstrated in a small‐scale Canadian trial, including 20 schools and 1492 children, run by the developers (Mishara & Dufour, 2020), who reported that children responded positively to the comic‐format sessions. Intervention schools outperformed control schools in terms of coping and social skills. Yet, there has been no independent review of the intervention. Furthermore, while the initial study included focus groups with students and teachers, reporting of the resultant data was extremely limited. Findings were not presented as qualitative themes, but more generally as positive statements narrowly demonstrating acceptability and feasibility of the intervention (Mishara & Dufour, 2020). Thus, while there is preliminary evidence pertaining to the social validity of the intervention, our understanding of other implementation‐related processes and outcomes remains limited (as is the case more generally; Proctor et al., 2011). Participant experiences of delivering and engaging with Passport, and the factors influencing these processes, remain unknown. As it continues to be delivered across schools in the United Kingdom, a rigorous, independent evaluation of student and staff experiences of the programme is timely.

### Implementation theory

Implementation is multi‐dimensional (Berkel et al., 2011), comprised of eight dimensions (Dane & Schneider, 1998): fidelity, dosage, quality, reach, responsiveness, adaptation, programme differentiation and monitoring control conditions (see Table S2). Aligned with Berkel et al.'s (2011) multi‐dimensional model, we conceptualize implementation as a superordinate construct, defined as how a programme designed by the developer differs from the programme delivered in practice (Domitrovich et al., 2008), and, thus, we explore student and staff experiences and perceptions of programme delivery across the various dimensions of implementation. Domitrovich et al.'s (2008) multilevel framework of implementation quality guides us in exploring factors influencing both implementation and of the support system necessary to sustaining it. According to this framework, factors influencing implementation occur across and between three hierarchical levels, categorized as individual‐level factors (e.g., implementer perceptions of and attitudes towards the intervention, and professional and psychological characteristics), school‐level factors (e.g., organizational determinants including characteristics and climate, administrative leadership and available resources) and macro‐level factors (e.g., community determinants including policies and community partnerships). This framework was developed when school‐based intervention implementation research predominantly conceptualized implementation according to delivery (i.e., dosage), and adherence to content and structure (i.e., fidelity), such that only teacher perspectives were evaluated. Less is known about children's perspectives, particularly across implementation dimensions of reach and responsiveness.

In Passport, teachers are encouraged to adapt the intervention during delivery to meet classroom needs (Mishara & Dufour, 2020; Partnership for Children, 2019). Exploring pupils' perspectives of and engagement with the intervention and their responses to adaptations is therefore crucial, particularly with recent evidence that children and young people can be attuned to mismatches between wellbeing provision and the school context (Demkowicz et al., 2023). Qualitative methods are best suited to capturing the dynamic processes at play during implementation (O'Conner et al., 2017). Exploring these complex processes underpinning the effectiveness of a school‐based intervention—in this case, Passport—within its social context is crucial to understanding ‘real world’ programme success or failure and its complexities (Greenberg et al., 2005; Nilsen, 2022).

Over and above furthering understanding of Passport specifically, our study advances understanding of school‐based intervention implementation processes and outcomes more generally. Research in this area has a longstanding emphasis on investigating internal validity of trial outcomes by evaluating whether a programme was delivered as the developer intended (i.e., with fidelity), conceptualizing poor implementation as deviation from content or structure (Durlak & DuPre, 2008; Humphrey et al., 2016). While a growing body of research is adopting a more multi‐dimensional approach, there remains a disproportionately greater focus on quantitative measures of fidelity and dosage, with research on reach, responsiveness and adaptation, particularly using qualitative methods, being notably absent for the most part (Durlak & DuPre, 2008; Rojas‐Andrade & Bahamondes, 2019). Where qualitative work is undertaken, this has often privileged teacher perspectives (e.g., Lendrum & Askell‐Williams, 2019), limiting our understanding. Qualitative research triangulating multiple perspectives within school‐based intervention evaluations can help to move beyond large‐scale patterns to understand the nuance in how different components connect to real‐world needs in the classroom, both generally as well as for specific contexts (e.g., transferability to England), subgroups (i.e., children recognized as vulnerable to social and emotional difficulties) and from varying perspectives, including children themselves. This is critical given evidence that implementation is a key moderator of effectiveness (Cipriano et al., 2023; Durlak et al., 2011). Thus, this study has the potential not only to develop understanding of Passport but also to guide ongoing development, adaptation and implementation of SEL interventions in English primary schools and to provide a methodological contribution to demonstrate how qualitative inquiry can contribute meaningfully to RCTs. Accordingly, our study addresses four key research questions:

1. What are the perceptions and experiences of school staff and children in delivering and engaging with Passport?
2. Is Passport implemented as intended by the developer?
3. What factors impede or facilitate implementation?
4. Where do the views of school staff and children converge and diverge in relation to the above?

## METHODS

### Design and team

The study is embedded in a two‐arm (intervention/control) parallel cluster RCT design, with 62 mainstream primary schools across Greater Manchester and the surrounding areas. Schools allocated to the intervention arm implemented Passport for Year 5 pupils (aged 9–10) in the academic year 23/24. Teachers received training in September 2023, and booster training approximately 3–4 months later. Child‐level outcomes were assessed at baseline (T0) and post‐intervention (T1).

The primary aim of the trial was to investigate the impact of Passport on children's internalizing symptoms and various secondary outcomes post‐intervention in implementing schools (compared to usual provision). This was operationalized through discrete concurrent strands expanding our inquiry by addressing more granular research questions centred around intervention effects, explanatory mechanisms, cost‐effectiveness and implementation and process evaluation. As such these strands were undertaken independently of one another, rather than directly informing one another (see Greene et al., 1989). We therefore report separately on the different strands at this time, rather than through a mixed integration process drawing together key conclusions.

The current study focuses upon the qualitative strand, exploring staff and student perceptions and experiences of implementing and engaging with Passport. It describes a longitudinal qualitative case study strand in five schools delivering the intervention, engaging with children, teachers and senior leaders. A case study approach allows contextually grounded examination across the delivery cycle, and triangulation of different parties' accounts. Approximately halfway through delivery (later stages of first term, 23/24), we visited to speak to some Year 5 children and any teachers delivering Passport about perceptions and experiences so far. After the delivery period (second/third term, 23/24) we visited again to speak with children, teachers and relevant senior leaders.

Our approach to data generation and analysis was guided by a framework we developed from key implementation conceptual work and reviews (Domitrovich et al., 2008; Durlak & DuPre, 2008; Nilsen, 2022): implementation processes (comprising dosage, fidelity and adaptation, quality, reach and responsiveness and programme differentiation); factors affecting implementation (at the programme, school and teacher levels); perceptions of impact (both universal and among specific groups); and sustainability (including continuation of the programme and of specific components and practices and of factors affecting sustainability).

We adopt a social constructivist stance, recognizing reality as socially constructed and, accordingly, integrating perspectives from varied groups. We are a team of researchers, including young researchers, with shared interests in child and adolescent development, mental health and SEL, and no vested interest in Passport. We are part of the wider team undertaking the RCT and are involved in other strands of the work in varying roles. Data generation and analysis in the qualitative strand was completed after the RCT's baseline survey and before the main intent‐to‐treat analysis. We reviewed and developed focus group procedures and materials with young researchers (Authors 7/8/9) to ensure these were age‐appropriate, engaging and sensitive. We adopted open research practices including trial registration (ISRCTN12875599; registered on 24 November 2022), registered report formats including for the current paper, pre‐registration (https://osf.io/pa46z/files/osfstorage/65ce8abfb74cac0297837a14), collaboration with young researchers, detailed methodological accounts and making data generation materials (Open Science Framework [OSF] link: https://osf.io/d6fgq/?view_only=f3c752a1825042be9ef516e6488cb393), data (via the OSF at end of the project, after December 2025) and coding (via Supporting Information) available.

### Recruitment and participants

#### Recruitment

Of the intervention arm schools, we identified and recruited five case study schools. This number offered scope for understanding patterns and variation and represents a reasonable proportion (one in six) of the overall intervention arm. To facilitate heterogeneity in need and context across schools, we identified and invited schools who varied in some combination of baseline level of internalizing difficulties and proportion of pupils eligible for free school meals (FSM), with additional attention where possible to varying school size and area of the region. We focused on baseline internalizing difficulties and FSM eligibility because these factors reflect both pupil mental health needs and broader resource pressures, which influence how schools engage with and implement Passport. Varying schools along those dimensions also captures differences in community context and school priorities, helping us understand how such factors shape barriers, facilitators and experiences across settings. We used FSM eligibility as a marker of economic disadvantage because it is the most reliable and practical measure currently available in UK schools, though we note limitations in its precision given, for instance, it does not capture the depth or duration of poverty, nor unregistered but entitled pupils (Siddiqui & Gorard, 2023).

**FIGURE 1:**
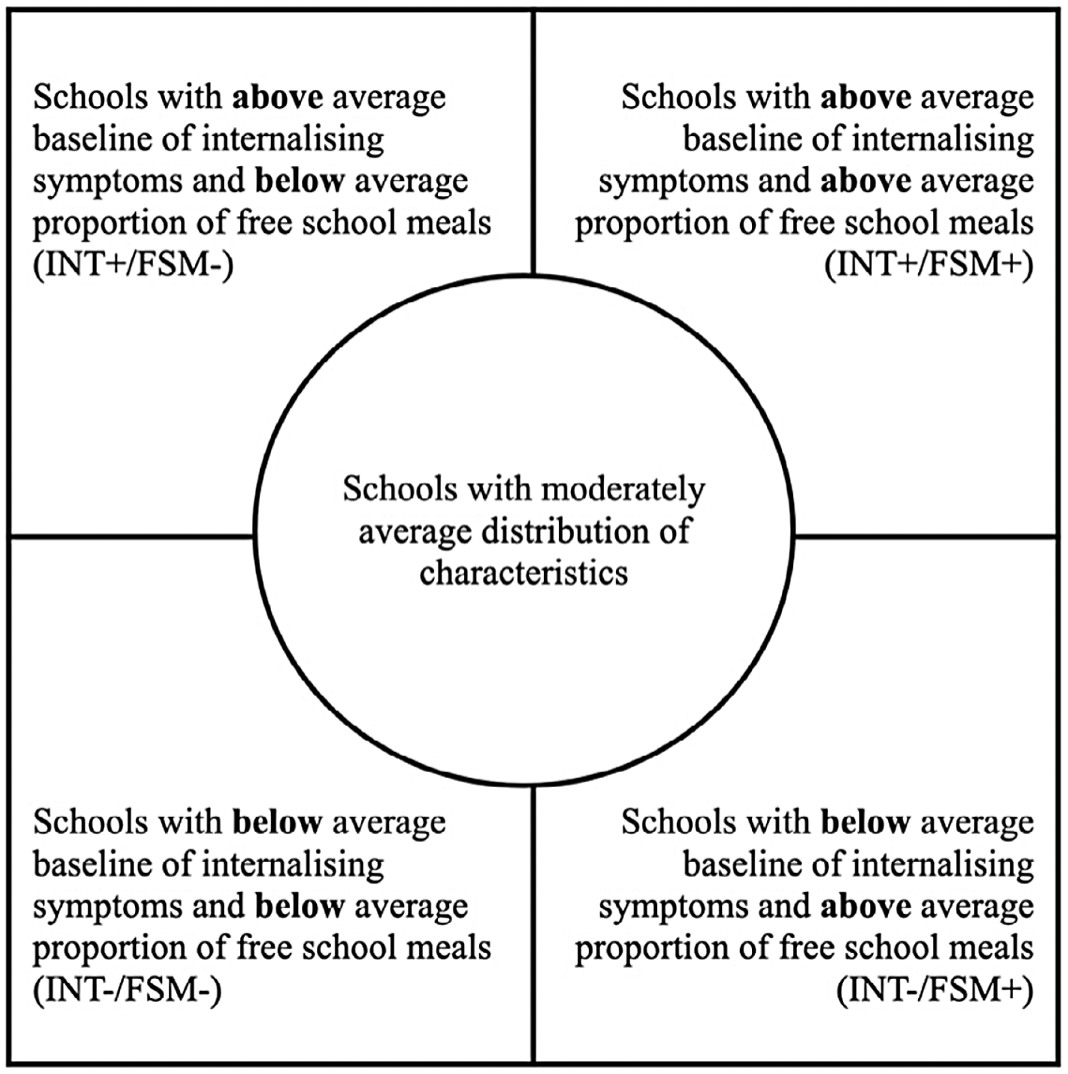
Visualization of categories of schools according to internalizing symptoms and proportion of free school meals.

Specifically, we mapped schools' spread across baseline difficulties and free school meal eligibility, categorizing schools into four quadrants capturing varying degrees of these characteristics alongside a fifth group with a moderately balanced distribution of characteristics (see Figure 1). Within all five categories, we rank‐ordered schools so that we see as much variation in school size and location in the region as possible, enabling us to move down the list and maximize heterogeneity as we invite schools. We wrote to the first three schools in each category inviting them to participate on a first‐come‐first‐served basis, and continued down the list as appropriate until we had one school per group.

When approaching schools, we shared our teacher information sheet and asked contacts to ensure that the teacher(s) implementing Passport were in principle willing to engage in case study work, to avoid later coercion or follow‐up loss. At Passport training, a researcher briefly outlined the case study approach and that we would be inviting involvement. Once case study schools were established, we asked staff to invite children to participate, emphasizing the importance of range in demographics (e.g., gender, special educational needs and disability [SEND]) and that we were not seeking to only speak to those who might be more articulate, well‐behaved or enthusiastic, following previous success using such strategies (Hennessey et al., 2021). We invited children via child/parent information packs, and established parent/carer consent and child assent; at this stage, parents/carers were asked to provide demographic information about their child (age, gender, ethnicity, free school meal eligibility, whether they have SEND, and whether they spoke English as an additional language).

#### Participants

We spoke with 51 children at visit 1 (across 7 focus groups, as two schools had two‐form entry) and 48 children at visit 2 (again across 7 focus groups). Of these children, we engaged with 30 girls (58.8%) and 21 boys (41.2%). For ethnicity, 23 participants (64.7%) described themselves as white, 11 as Asian (21.6%), 3 as mixed or multiple ethnic group (5.9%), 2 as Black (3.9%) and 2 as another ethnic group (3.9%). Nine children (17.6%) were or had been eligible for free school meals, 1 (2%) had SEND, and 7 children (13.7%) spoke English as an additional language. We also engaged with 7 teachers and 4 senior leaders (we were unable, in one school, to engage a senior leader).

This surpassed, for children, our initial estimated sample size. We noted this as sufficient for in‐depth analysis given the focused nature of discussions (Malterud et al., 2016). We aimed to have 5–6 children in each group, but over‐recruited by inviting 8 per group, accounting for non‐response, non‐attendance or non‐assent.

### Data generation procedures

#### Focus groups with children

Focus groups occurred in a private school area, led by two researchers. We reviewed a visual group agreement to set expectations and address social dynamics (e.g., emphasizing honest perspectives, clarifying our independence from Passport, confidentiality boundaries). We used a semi‐structured guide (https://osf.io/d6fgq/?view_only=f3c752a1825042be9ef516e6488cb393), with questions and prompts designed to examine relevant aspects of our conceptual framework, namely: reach, responsiveness, quality and perceived impact. For instance, we asked children, *Think back to the first time you did a Passport lesson. How did you feel about it?* with follow‐up prompts asking how they feel about it now and how their teacher appears to feel during delivery, to explore responsiveness and quality dimensions. We shared child‐facing Passport resources to aid specificity and embedded creative strategies and physical props to facilitate engagement, candour and inclusivity (Hennessey et al., 2021). For instance, children were invited to write what they do/do not like about Passport on post‐it notes and add them to a green bucket and a red bucket, respectively, to examine together; at the second visit, we introduced ‘magic wands’ to aid solution‐focused discussions. Focus groups lasted 39–77 min (mean = 54 min, 34 s).

#### Interviews with teachers and senior leaders

School staff interviews occurred either in a private school space or over Zoom. We shared information sheets before the first interview and established informed consent. We reminded staff of our independence from Passport to facilitate candour and clarified that school colleagues may recognize their data.

We followed a semi‐structured schedule (https://osf.io/d6fgq/?view_only=f3c752a1825042be9ef516e6488cb393) focused on unpacking specific theoretical constructs across the full conceptual framework. Teacher interviews focused more on delivery and classroom context (e.g., *What do you think are the similarities and differences between Passport and other wellbeing initiatives or practices in which the school has been involved?* to explore programme differentiation), whereas interviews with senior leaders triangulated this and included greater focus on wider school context/strategy (e.g., *If other schools were to deliver Passport, do you think there's anything they should know about how to do it well?*). For teachers, we brought Passport resources to facilitate specificity (e.g., teacher guide, session resources). Staff interviews lasted 20–49 min (mean = 32 min, 22 s).

### Ethical considerations

The project, including the qualitative strand, was approved by The University of Manchester's University Research Ethics Committee (ref: 2022‐14050‐24401). For the focus groups and interviews, parental consent and pupil assent were obtained in advance of focus groups beginning, with clear child‐friendly information, a letter encouraging discussion at home, and continued monitoring of signs that children may not genuinely wish to take part. Case study schools were asked to check that key colleagues were in principle interested in signing up to this strand of the RCT to avoid later pressure for them to engage in interviews, and teachers were made aware that their participation might be identifiable despite anonymization given the case study nature of the engagement. Researchers managed focus group dynamics carefully, set clear boundaries and maintained confidentiality, while all data were securely stored and both school‐based and institutional safeguarding protocols followed.

### Data analysis plan

We used reflexive thematic analysis (RTA), to analyse patterns and nuances with recognition that researchers shape analysis (Braun & Clarke, 2006, 2021). Our analysis sat across the inductive to deductive continuum, guided by a framework we developed from key implementation conceptual work and reviews (Domitrovich et al., 2008; Durlak & DuPre, 2008; Nilsen, 2022). As in Table 1, these function as pre‐identified themes (deductive), within which constructed major codes reflecting participants' perceptions and experiences within these areas (inductive). For instance, the implementation over‐arching theme includes implementation dimensions such as ‘fidelity and adaptation’, which was developed to comprise major codes capturing perceptions and experiences of adaptations.

**TABLE 1:** Planned thematic structure.

Deductive over‐arching theme	Deductive sub‐theme	Inductive major codes to be determined
Implementation processes	Dosage	
	Fidelity and adaptation	
	Quality	
	Reach and responsiveness	
	Programme differentiation	
Factors affecting implementation	Programme characteristics	
	School factors	
	Teacher factors	
Perceptions of impact	Perceived universal impact	
	Perceived impact among specific groups	
Sustainability	Continuation of programme	
	Continuation of specific components/practices	
	Factors affecting sustainability	

Analysis was facilitated using NVivo. We familiarized ourselves with data, then engaged in line‐by‐line coding, organizing codes under thematic areas. In initial stages, analysts (Authors 2/3/4/5/6) focused on data from case study schools they each led on, facilitating a contextually grounded analysis. Next, analysts came together, joined by Author 1, to construct major codes reflecting key patterns. We reviewed and refined major codes (checking for coherency, distinctness, and reflection of the dataset) and collectively defined and named them.

Data from all sources were analysed collectively to build an integrated picture (RQs 1/2/3). Once complete, we triangulated and examined convergence and divergence in thematic representation among groups (RQ 4; children, teachers, and school leaders). We contextually examined associated data and coding for major codes for convergences, differences or nuances on the same aspect among groups, using NVivo's code‐tracking function to examine representation of data sources within codes. We present this cross‐group analysis within our thematic write‐up.

We attended to quality guidance (Braun & Clarke, 2021) and undertook peer‐debriefing throughout analysis, including consulting with young researchers (Authors 7/8/10) to refine interpretation. Use of multiple analysts was not to establish coder reliability but, aligned with RTA, to support reflexivity and closeness with case study contexts.

## FINDINGS

Table 2 outlines case study school characteristics: type, form entry size, positioning within RCT schools in terms of baseline difficulties and free school meal eligibility and reflections on overall intervention engagement.

**TABLE 2:** Case study school characteristics.

School ID	School type	School size	Baseline int. difficulties (relative to sample)	FSM eligibility (relative to sample)	Reflections on overall engagement (from each case study school's lead researcher)
School A	Community school	Two‐form entry	Higher than average across participating schools	Lower than average across participating schools	Overall, both teachers and students responded positively to Passport. Some teachers observed that certain situation cards did not resonate with students from higher socio‐economic backgrounds. Consequently, they preferred using blank situation cards, enabling students to create scenarios that felt more personally relevant. One teacher also noted the need to adapt several sessions to align with the busy and sometimes dynamic Year 5 schedule. For instance, during Ramadan, he shortened sessions to accommodate students who were fasting. The school's open‐classroom design occasionally meant noise from adjacent classes filtered in, but teachers reported that students were accustomed to this and that it did not diminish engagement.
B (6)	Community school	One‐form entry	Lower than average across participating schools	Lower than average across participating schools	The teacher reported enjoying delivering Passport and thought it was an appropriate fit for the class, and children described enjoying Passport and seeing value in it. However, the teacher noted being unable to complete the programme, reportedly due to competing priorities such as transition days, school trips and Ofsted inspections, and the children and teacher reported skipping sessions or just skimming over content and combining sessions. The teacher also noted struggling with strategies to manage some pupils' emotional reactions to the content, and children too noting that the teacher could have handled some situations better. This included sensitivity in handling bereavement content in the session, which the teacher reported not knowing how to handle while also managing the full class. Note that, in this school, we were unable to establish an interview with a senior leader.
C (7)	Community school	One‐form entry	Lower than average across participating schools	Higher than average across participating schools	The school's relationship with Passport appeared positive overall, and the teacher and most children appeared to enjoy Passport. The teacher appreciated its adaptability to their needs in a disadvantaged area, especially in helping children express emotions they might not learn at home but wanted more hands‐on learning opportunities, such as seeing a lesson in action or connecting with other schools using Passport. Though overall children reported enjoying Passport, some students reported finding it boring.
D (10)	Voluntary‐aided religious school	One‐form entry	Average in the context of participating schools	Average in the context of participating schools	The teacher reported that they loved delivering the Passport programme mainly because the children enjoyed it. The children liked the lessons and described practical examples of when they used their newly acquired coping skills including outside of the school setting. Staff described that the school was situated in a community with high levels of domestic violence and little migration from the town, and that experiential learning was a key priority. There were many universal and targeted social, emotional and mental health initiatives in the school, which had a noticeably warm and supportive atmosphere. Some created new characters for the programme to capture diverse individual needs and differences (e.g., a lonely character, a neurodiverse character). Due to competing responsibilities, the teaching assistant delivered a number of the Passport lessons to ensure complete delivery.
E (15)	Voluntary‐aided religious school	Two‐form entry	Higher than average across participating schools	Higher than average across participating schools	School leaders, teachers and children appeared to like Passport and described a positive engagement with it. Both teachers spent time adapting lessons to fit the school's expectations and norms but were enthusiastic about it and proud of their adaptations. This included additional created resources, many of which seemed focused on evidencing learning. Teachers felt that there was a high need for additional support among children and their families and appeared to value social and emotional learning as part of a wider holistic approach.

*Note*: Baseline difficulties and FSM eligibility are relative to the overall sample of participating schools (rather than national average) to illustrate their positioning specifically within our recruitment strategy as outlined in Methods.

Abbreviations: FSM, free school meals; Int., internalizing.

In Table 3 and below, we present the major codes within each of our predetermined deductive theoretical themes and subthemes.

**TABLE 3:** Overview of major codes constructed within each deductive theme.

Deductive over‐arching theme	Deductive subtheme	Inductive major codes
Implementation processes	Dosage	All Passport lessons were delivered in most cases Lessons were not always delivered weekly
	Fidelity and adaptation	Classifying and prioritizing activities based on their perceived importance Use of lesson materials varied across time and between classrooms Activity timings were a guide, not a rule Lessons adapted or augmented to produce written work Changes made or planned in response to perceived or actual pupil need or engagement
	Quality	Additional time spent planning or personalizing Passport for my class Using knowledge of the class to deliver content mindfully and appropriately
	Reach and responsiveness	Enthusiasm, enjoyment and excitement around Passport lessons Children are relaxed and engaged compared to traditional lessons All children have a voice and a say during Passport lessons Some children get upset by during serious topics
	Programme differentiation	Broadly similar, with slightly wider range of topics, to wider content in other SEL programmes and PSHE (Personal, Social, Health and Economic) education resource Some greater breadth and relevancy in content More engaging for children than other materials Less burdensome for teachers Complementary component of wider PSHE and whole school provision
Factors affecting implementation	Programme characteristics	Navigating too much time and too little Engagement and child friendliness Relevance in the classroom Optimizing programme delivery and resource use
	School factors	Alignment with current practices and needs Impact of student and family background on implementation School environment and leadership impact on implementation
	Teacher factors	Perceived delivery and resource navigation Teacher buy‐in and commitment to
	External factors	Training perceptions and recommendations
Perceptions of impact	Perceived universal impact	Improved emotional literacy Improved management of negative emotions such as anger, grief and loss Helped children manage and cope with bullying Improved social and relationship skills Helped children know they can seek help Helped children increase repertoire of coping strategies, although sometimes these were not helpful Improved children's relationship with teacher
	Perceived impact among specific groups (though not mutually exclusive)	Greater benefit for children with social, emotional and mental health difficulties Boys and girls benefitted in different ways because they had different needs Children from ‘disadvantaged’ homes benefitted more (more need) Helped lower attaining pupils with emotional literacy but they sometimes struggled Helped children with English as an additional language with emotional literacy Helped teachers manage their own emotions
Sustainability	Continuation of programme	Openness to continuing programme with future year groups
	Continuation of specific components/practices	Teachers planning to continue using specific resources and ways of engaging with children in day‐to‐day practice
	Factors affecting sustainability	Primarily interested in a more extended and embedded version of the intervention

### Implementation processes

#### Dosage

Almost all teachers reported delivering all lessons, though one noted skipping or combining content which they attributed to wider pressures such as trips or Ofsted visits.

#### Fidelity

Teachers reported fidelity: ‘we have followed it really by the script’ (School A) but all simultaneously described some modification. Some did not adhere to timings: ‘usually we get 45 minutes, so we have to cut it down’ (School A), whereas others ran over: ‘because the children were getting so much out of (it)’ (School E).

Teachers described prioritizing and adhering to activities perceived as most important while reducing/removing introductions, conclusions, games and parent worksheets as if supplementary: ‘The main activity, yeah, that's the bit that I wouldn't change. That's like set in stone … if we don't do the evaluation […] they've still learnt the main part’ (School A).

Modifications were based on knowledge of children and to encourage engagement: ‘I know what my class are like and I'll tailor it to them… they're too grown up for [the game]… we often leave that out and have discussions instead’ (School A). Changes were both pro‐active and reactive: ‘Sometimes when they just sit there and it's all discussion… they just maybe lose focus’ (Teacher, School C).

#### Quality of delivery

Students picked up on teachers' attitudes towards Passport, describing them as ‘joyful’ (School D) or ‘bored’ (School C): ‘[teachers] feel happy [during Passport]… because we're open about our feelings’ (School B).

Some teachers spent additional time preparing and personalizing lessons:

I'd target [lower ability children] like you would any other lesson, those children that you think ‘Ooh, I'll just make sure that they have got it’ or I'll discuss with them. We did a lot more mixed ability groups or pairs. (School D)

Knowledge of students helped teachers deliver sensitive content mindfully: ‘One of my little girls just only a few years back lost her dad…’ I said, ‘If you don't want to contribute […] that's fine’ (School C).

Teachers sometimes prepared additional/alternative tasks to further embed learning: ‘rather than one big friendship tree collectively, they did their own personal ones… so they could personalise’ (School C). This also facilitated physical evidence for Ofsted inspections: ‘it should be we're trusted to do our job well but unfortunately, there's usually someone that's coming in asking’, ‘Well, how can you prove that you've done this?’ (SLT, School E).

#### Reach and responsiveness

Children largely exhibited and described enthusiasm and enjoyment during Passport: ‘whenever Miss says’, ‘Alright, we're doing Passport,’ I just want to jump out my chair and shout out really loud, ‘Hell yeah!’ (School D). Passport was described as distinct from traditional lessons: ‘it's quite visual, there's lots of talking, there's not that much writing involved and when it is, it's sort of anxiety free, it's their own little books so no‐one's going to come and mark it’ (Teacher, School D). Most described feeling relaxed and engaged: ‘It probably felt easier because… say before that we'd probably do like writing or something like that. But we don't really in Passport do writing that often’ (School A).

Teachers spoke of reduced absenteeism on days Passport was scheduled: ‘one of the children who's struggling to come into school… I said, ‘Oh, we're doing Passport this afternoon. Would you like to come in? And he has done’. (School D). Children who typically did not contribute in traditional lessons were more vocal: ‘there's a few girls in there who you might not normally hear from… they were becoming more and more confident speaking about [emotions]’ (Teacher, School B). However, incidences of children becoming upset during discussions of grief and loss were described in three schools: ‘people were crying so she had to take them out and calm them down’ (Child, School E).

#### Programme differentiation

Teachers and SLT noted that Passport covered similar topics and objectives to other SEL/PSHE programmes. Some suggested Passport covered a slightly broader—if not fully comprehensive—skillset, and was, ‘more relevant […] for instance, when it was all about a friend leaving’ (Teacher, School E). Passport's loss and bereavement content was particularly noted as less common in other programmes. Teachers noted that materials were more engaging than other approaches—especially the comic book format—and commended ready‐to‐use materials: ‘it's very easy to access for us as teachers and for the children to take part in’ (School C). Teachers, SLT and children positioned Passport as one complementary component of PSHE and whole school provision, suited to their ethos and interconnected with other approaches (e.g., breathing exercises in other sessions), and that varying programme content facilitated a comprehensive whole (e.g., with Passport not covering puberty, which other programmes did).

### Factors affecting implementation

#### Programme characteristics

##### Navigating too much time and too little

Some teachers felt lesson length was appropriate: ‘any shorter, it would have maybe lost a little bit of its impact, and I don't think it needs to be any longer’ (School D). However, perspectives varied. Others found lessons too long to hold attention, especially on mornings; others felt material was hard to fit in: ‘that's a long time for me to be stood up teaching and them responding. Like they will get bored, no matter what you're talking about’ (School A). Some suggested more variety or less material. Children described challenges finishing tasks on time.

##### Engagement and child friendliness

Passport was praised for helping children engage with serious topics in a child‐friendly, accessible, and enjoyable way. Teachers highlighted the role of interactive, well‐sequenced activities; children noted the difference from traditional schoolwork, though some disliked excessive talking.

##### Relevance in the classroom

Overall, teachers felt sessions suited class needs: ‘a lot of the lessons that come through and a lot of the topics were very relevant’ (Teacher, school 6).

##### Optimizing programme delivery and resource use

Though some found the master folder clear and the structure helpful, others wanted clearer guidance, including additional examples and explanations. Teachers and children desired a wider activity variety, especially for facilitating emotion expression. They emphasized boundaries during sensitive discussions, as some teachers struggled when children shared private information. Children desired reduced teacher‐led explanations and more independent activities, particularly for sensitive topics: ‘you might just want to talk to… like in front of the whole class, you might just write on a paper and give it to your teacher’ (Child, school 7). Some children found Passport, especially animations, repetitive and slow, leading to disengagement.

#### School factors

##### Alignment with current practices and needs

Teachers felt Passport aligned with school priorities, particularly around emotional well‐being, SEL and personal development. Several noted that it was straightforward to schedule, often replacing PSHE lessons, though teachers and SLT noted that Passport required adjustments and added to workload: ‘it is difficult to get it in’ (Teacher, School 10); ‘[teachers were not] massively happy because we have […] time constraints’ (SLT, School 15).

##### Impact of student and family background on implementation

Most teachers noted that Passport could be tailored to meet distinct needs. Children's background and family circumstances (e.g., at‐home emotional expression context) influenced implementation, meaning some topics needed more focus or expansion.

##### School environment and leadership impact on implementation

Teachers highlighted that SLT support, delegated flexibility with timetabling, and positive teacher relationships were crucial for engagement. They noted physical space was needed for storing resources and to move around for activities.

#### Teacher factors

##### Perceived delivery and resource navigation

Some teachers expressed dislike for Passport and would not recommend it, noting a need for streamlining: ‘I wouldn't mind having a go at a better version’ (Teacher, School 3). Conversely, some found it engaging and beneficial: ‘I've liked teaching it, I've thought it's been really good, really good fun for the children, so… there's nothing really that […] I'd change’ (Teacher, School 15).

Teachers desired greater autonomy. Both teachers and children highlighted classroom relationships as facilitating delivery and engagement (‘our relationship with the teacher is really good’; Child, School E). Teachers suggested that less experienced teachers may need extra time for adaptations.

##### Teacher buy‐in and commitment

Teachers noted SEL's importance, and some noted that Passport could be ‘eye‐opening’ (School E). SLT reinforced the need for teachers' buy‐in: ‘you need to get the teachers to buy into it if you want to do it well […] to understand why it is that we're doing this and how important it is’ (School E). Children suggested that teacher enthusiasm was key; disengaged or ‘bored’ teachers potentially diminished interest.

Children expressed responding well to teachers who were kind and emotionally responsive, which could help engagement: ‘I would teach it like [teacher], she's amazing with trying to help us cope with our emotions’ (School E).

#### External factors

##### Training perceptions and recommendations

Many teachers recommended more tailored training, including visual materials and real‐life examples. Teachers suggested that communication with other schools could improve understanding and implementation, including, for instance, a peer support forum.

### Perceptions of impact

#### Perceived universal impacts

##### Improved emotional literacy

Children reported Passport as useful for emotional expression and help‐seeking, while teachers observed greater expressiveness and vocabulary: ‘rich discussion around emotions has been really useful for them […] it's given them maybe a little bit more vocabulary and that nuance’ (Teacher, School D). Children described better understanding others' emotions, feeling enabled to support friends, and reassured by shared emotional experiences: ‘I thought I was the only one who was sad, so after realising there was people, I was really happy we did it’ (School D).

##### Learning different coping strategies

Children provided anecdotes of coping strategies learned, including in ‘everyday’ situations (e.g., sibling/peer disagreements), and more acute experiences (e.g., loss of friends, deaths of pets and family). Children described learning new ways to cope with and manage difficult emotions, like anger, in and beyond school, which they and teachers felt supported interactions and academic challenges:

I'll be in the playground, someone will have kicked me or shouted at me, and I want to scream at them. But then my brain sparks… ‘Hey, you remember that Passport thing? You need to listen to that thing, or you might get a friend deleted’. (Child, School D)

Children and teachers thought it helpful that Passport encouraged children to explore strategies that worked for them, instead of as a whole ‘in school we cope like this…’ (Teacher, School A). Children generally believed Passport would help them cope with future situations, like exam stress, making new friends and the primary‐to‐secondary transition. Teachers said that they were usually cautious about ‘big’ topics like loss/grief, but that coping‐focused content bolstered confidence.

##### Less helpful elements of Passport

Teachers felt some children struggled to ‘make the link between what they learnt in [Passport]’ and everyday life (School D). Bullying was salient for children, and while they said Passport helped them cope with victimization through learning ‘not to listen to bullies’ (School E) and ‘start speaking up’ (School A), they felt Passport did not stop bullying.

Children found some lessons upsetting; reflecting on challenging experiences reminded them of difficult emotions like anger or sadness:

Thinking about happy times with the person you've lost or like your pet… it just makes me even more upset… it makes me want to go up to my room and… just sit in silence in my bed hugging my favourite teddies. (School D)

Teachers noted that loss content ‘affects [children] quite hard’ (School B). Children reflected that how teachers managed upset was important, for instance, going ‘outside for a little talk’ or offering a ‘warm hug’ (School E) helped.

#### Perceived impact among specific groups

Teachers identified subgroups they thought had benefitted in particular ways, seemingly grounded in wider assumptions of needs.

##### Gendered differences

Girls were viewed as ‘more mature’ than boys, with fewer friendship problems, but also as embarrassed by feelings and having more ‘anxiety’. Teachers believed girls gained particular benefit from ‘talking about emotions’ more confidently and ‘processing emotions’ (School B). Teachers described boys as ‘struggling’ more with anger and frustration; Passport reportedly helped boys recognize anger and ‘deal with it in a more positive way’ (School B).

##### Children from ‘disadvantaged’ households

Teachers suggested Passport was especially beneficial for children they identified as being from ‘disadvantaged families’ with ‘difficult home lives’. Teachers believed that these children had greater support needs, particularly with regulation, anger and ‘lashing out’ and low self‐esteem and confidence. Teachers reported lowered problematic behaviour since starting Passport: ‘they get [more and more] upset […] whereas now […] you don't really see that’ (School E).

Passport was considered less helpful for multiply disadvantaged children, for instance, where attainment intersected with social and emotional mental health (SEMH) needs, ‘difficult home lives’ and trauma. Teachers thought these children struggled more to apply Passport to their lives and needed further intervention/support (School D).

##### Children with additional needs and lower attainment

Teachers expressed frustration that Passport was not differentiated for children with SEND, lower attainment and children with English as an additional language. They adapted activities and materials, ‘targeting and identifying what they need from that resource’ (School E), and where sessions allowed this teachers reported emotional literacy improvement: ‘they couldn't just process… their emotion, and now they can tell me things like they just feel tired throughout the day, they feel a bit alone’ (School C). Where activities were not adaptable, pupils with varying needs could not participate fully or experience the same learning opportunities: ‘there's nothing for your SEND kids… if it's a discussion […] what he can get out of or contribute to discussion is… fairly minimal… but in any other lesson, you wouldn't do that’ (School A).

### Sustainability

#### Continuation of programme and of specific components/practices

Several teachers and SLT were open to using Passport again, describing it as useful for children and straightforward and/or enjoyable to teach. Some suggested that it could be a targeted programme for those with emergent difficulties; others would consider extending to other year groups, such as Year 6 children preparing for transition:

I really like the idea of Passport to Success being an intervention programme for those difficult times and it might be that they follow the programme… explicitly or it might be that they dip in and out depending on the situation or the needs. (SLT, School‐E)

Some teachers drew attention to specific practices or resources they were interested in continuing more generally, particularly as a tool for talking with children, such as ‘golden rules’ for talking about an incident, or characters to talk about feelings.

#### Factors affecting sustainability

When reflecting on potentially implementing Passport again, teachers and SLT focused less on facilitators or barriers, and generally noted a desire for Passport to be a more immersed or integrated approach rather than a ‘standalone’ fixed‐term intervention. Several liked the tools it provided for language or felt it had potential benefit beyond Year 5 and expressed interest in a version more comprehensively embedded into school life: ‘Something that could be more immersed for multiple year groups […] but not as a standalone’ (Teacher, School A).

## DISCUSSION

We examined implementation processes for Passport in English schools, exploring perceptions and experiences of staff and children. Here, we explore Passport as a case through which to advance theoretical understanding of how school‐based SEL interventions are interpreted, adapted and enacted in practice, particularly in relation to fidelity, differentiation and children's sense‐making.

Generally, teachers reported delivering Passport to the expected dosage and described effort to deliver as intended while making adaptations they considered appropriate. Overall, Passport was seen as engaging, age‐appropriate and useful for emotional literacy, but encompassed challenges including preparation time, sensitive topics and limited in‐built differentiation for additional needs (Note: we refer in this discussion to two distinct forms of ‘differentiation’: *programme differentiation* [how an intervention differs from others] and *instructional differentiation* [how curricula are adapted to meet varied classroom needs]).

Children, teachers and SLT were generally aligned in perspectives where overlap in insights existed, except in one school where a teacher perceived low enthusiasm among children, while children reported high enthusiasm (possibly due to misunderstanding between these parties *or* perceived social desirability towards researchers). However, within‐group differences became evident; for instance, teachers varied on ease of delivery in time limits, and though most children enjoyed Passport, some found content repetitive and slow. Given the varying teacher and school factors affecting implementation (including alignment with school context, leadership impact, perceived resource navigation and teacher buy‐in), it is likely that such factors partially shaped this, alongside factors like teacher experience. This cross‐group convergence strengthens confidence in core implementation mechanisms, while within‐group divergences illuminate the ways in which individual psychological, professional and contextual factors shape enactment and experience.

### Training, fidelity and adaptation

Our findings contribute to implementation theory by demonstrating how fidelity is not a fixed construct but is actively interpreted by teachers through personal pedagogical logics. Passport is manualized to ensure fidelity, with strategic personalization to improve contextual fit (Mishara & Dufour, 2020; Partnership for Children, 2019). Despite self‐reporting high fidelity in surveys (Humphrey et al., 2025) and interviews, teachers simultaneously described adaptations, ranging from teacher‐focused (e.g., anecdotes) to student‐focused (e.g., need differentiation; Lovett et al., 2024), alongside augmentations and modifications. While augmentations can facilitate engagement (Lendrum et al., 2016), modifications can compromise change mechanisms if removing/changing core components (Hansen et al., 2013)—for example, ‘Dragon's Path’ (an interactive game played at the end of sessions in which children apply strategies they have learned to overcome difficult situations along the dragon's path). This confirms positivity bias in self‐reported fidelity (Hansen et al., 2014) and differing interpretations of fidelity between teachers and researchers, echoing Zetterlund et al. (2022) on differing understandings of adaptations (‘some adaptations do not count’; p. 5). Importantly, this suggests that commonly used fidelity measures may capture teachers' *intentions* rather than their *operational definitions* of core components, with implications for how fidelity is conceptualized and measured in SEL and, more broadly, in educational psychology. Understanding such conceptualizations is essential, particularly given calls for improved SEL fidelity measures (O'Brien et al., 2025).

Excluding core components may reflect poor implementation support, including training (Domitrovich et al., 2008). Partnership for children delivered training differing in content, structure and length to the Canadian trial (Mishara & Dufour, 2020). This included endorsing and encouraging adaptations, without explicit instruction on core components. Although booster training was introduced to ensure adherence to essential ingredients, attendance was poor (18%). Additionally, Partnership for Children does not offer the consultant support available to Passport schools in Canada (https://www.passeportsequiperpourlavie.ca/language/en/profile/teachers/), noteworthy given teachers' desire for personalized support and pedagogy‐focused training.

### Lessons from passport programme differentiation

An important contextual consideration is that since completing this qualitative analysis, our intention‐to‐treat analysis within the wider trial (reported on in Humphrey et al., 2025) revealed null effects for Passport across all measured outcomes. Our review of explanations in interpreting that intention‐to‐treat outcome has, based on qualitative data insights as well as implementation and business‐as‐usual data, ruled out implementation failure (where key aspects of an intervention are delivered substantially differently to as intended), programme theory failure (where intended mechanisms do not operate as assumed) and research failure (where study design limits reliability of findings). However, insights across data points *did* highlight differentiation failure (where the intervention is not sufficiently distinct from usual practice, limiting its ability to show additional effects) as a potential explanatory factor for these null effects, given evidence of widespread use of proprietary SEL interventions and substantial content overlap between Passport and usual provision (Humphrey et al., 2025).

Some features distinguished Passport from other SEL content to participants, however, suggesting that perceived distinctiveness and measurable differentiation do not always align in complex school environments. Its comic book format was considered engaging, age‐appropriate and refreshingly different from typical lessons; notably, such an approach creates a continuous narrative world that combines visual and textual storytelling to engage children in longitudinal, immersive learning, which is distinct from many standard SEL narrative approaches. Comic books have been explored in educational and medical contexts, and recent literature and our findings suggest that they offer promise in SEL by visually representing emotions in an engaging way, creating a safe world for exploring feelings (e.g., Zagkotas, 2025). The use of comic books and similarly rich narrative approaches may be an important mechanism for further exploration in SEL and wider PSHE curricula. In reflecting on Passport's impact for bolstering coping strategies, teachers and children foregrounded the individual psychological importance of structured reflection regarding how and when coping strategies might work for them. This design feature of space to reflect upon the coping strategies being learned and their personal fit is rarely prioritized in SEL curricula and may offer a critical mechanism for supporting individual agency and coping flexibility. This is perhaps especially useful on the cusp of early adolescence, where individuals typically begin greater strategy differentiation (Zimmer‐Gembeck & Skinner, 2016). This appreciation of coping flexibility from both our child and teacher particiapnts highlights children as active agents in meaning‐making, extending educational psychological models of SEL that often prioritize adult perspectives on skill acquisition and overlook children's agency and individual differences.

Inclusion of topics like grief and loss stood out to teachers. Researchers have noted a scarcity of such content in SEL/PSHE curriculum, despite potential benefit (Dawson et al., 2023). Teachers' familiarity with pupils, setting of warnings where appropriate and relational qualities including empathy and responsiveness were referenced as central to navigating content in a manner that safeguarded children while supporting emotional learning. Findings suggest delivering such content well requires sensitivity, attention to individual needs and delivery flexibility. SEL programmes covering such topics may benefit from detailed training on handling content, facilitation reminders and optional private or flexible activities. These findings extend models of SEL by showing how sensitive content is mediated through relational trust and teacher judgement, highlighting the complex processes that shape emotional learning beyond standard curricula. Future research should consider how such topics can be designed and facilitated to limit potential harm and support positive outcomes, with consideration of psychological, relational and contextual circumstances.

Collectively, and in the context of our wider RCT outcomes (Humphrey et al., 2025), our qualitative findings extend existing accounts of differentiation failure by demonstrating a tension between evaluative distinctiveness and perceived pedagogical value. That is, Passport was, we believe, insufficiently differentiated from usual practice to generate additive effects, yet was experienced by many teachers and children as engaging and more personalized, and as including greater engagement with more sensitive topics that teachers can otherwise feel cautious to engage in. We have, accordingly, followed up with additional interviews with school staff focused specifically on whether and how practitioner perceptions of value are created in practice, which have highlighted that the costs and benefits of an intervention like Passport are not objective or static but constructed differently across different stakeholders and fluctuate over time (O'Brien et al., under review).

However, some teachers commented on Passport's classroom‐boundedness, desiring more embeddedness into school life, reflecting complex systems theory principles around how interventions can be designed to interact with and disrupt existing systems (e.g., Moore et al., 2018). This highlights opportunities to build SEL programmes that more fully interweave with whole school contexts, echoing previous suggestions that pushing beyond discrete interventions to build coherent whole‐school programmes is critical (e.g., Demkowicz et al., 2023).

### Differentiation opportunities

Teachers were clear that Passport, like many SEL interventions, does not inherently accommodate children with additional contextual needs—for instance, SEND (Hamilton et al., 2025, 2026). Approximately 18% of pupils in England have SEND, with speech, language and communication needs most prevalent (Department for Education, 2024). High cognitive load in SEL programmes, particularly content demanding attention or literacy, can hinder effectiveness (Shi & Cheung, 2024). Here, Passport's demands—particularly its reliance on group discussions—often require teacher adaptation of content *and* activities to foster SEND inclusion. Additionally, while teachers felt that children with EAL and lower attainment showed gains in emotional identification, they may have missed out on broader benefits.

Evidence on SEL outcomes for children with SEND remains limited. Few studies report number of participants with SEND (Cipriano et al., 2023), and some exclude children on this basis (Fu et al., 2024; Petras et al., 2008). Some trials indicate promise: children with learning disabilities have shown greater reductions in relational victimization (Sullivan et al., 2015) and improvements in readiness to learn and self‐regulation (Hart et al., 2020). Our findings emphasize that inclusive SEL requires consideration of how programme design interacts with individual cognitive and linguistic capacities, extending understanding of mechanisms underpinning differential effectiveness.

As children with additional needs such as SEND, EAL and lower attainment face increased risks of difficulties (Leekam, 2016; Ros & Graziano, 2018) and typically lower social and emotional skills (Carnazzo et al., 2019; Ralić & Marković, 2024; Thomson et al., 2017), making SEL interventions accessible is vital. To facilitate social inclusion for all children, teachers need support in providing collaborative learning opportunities (Broomhead, 2018; Lindner et al., 2022). Although some may benefit from targeted or multi‐tiered approaches, mainstream inclusion should remain a priority and is contradicted by intervention designs fostering exclusionary practices.

Findings highlight that intervention developers must recognize that ‘in schools, variability and diversity are the norm, not the exception’ (Cipriano & McCarthy, 2023, p. 5) and assess whether the structure and demands of SEL activities disadvantage specific groups. Developers should co‐design interventions with children with additional needs to enhance relevance and acceptability (Cullingham et al., 2024). To mitigate undue impacts of differentiating instruction on already pressured, time‐poor teachers (Pozas et al., 2023), guidance and support are necessary to facilitate fidelity without compromising inclusion.

### Strengths and limitations

Triangulating child, teacher and SLT perspectives provided a multidimensional picture of implementation, revealing cross‐group convergence and within‐group variation, and our theoretical framework enabled attention beyond fidelity to include adaptation, reach and responsiveness. Case study schools were purposively selected for contextual diversity, enhancing relevance, though not all school types were represented (e.g., no academies engaged). Though children were diverse in terms of gender and ethnicity, we had lower representation of those eligible for free school meals and those with SEND. The latter is an important limitation given teachers' accounts of the intervention as challenging for those with SEND.

Focus group dynamics can, of course, influence the narratives explored by participants. Our use of strategies to reduce social desirability (linked to ourselves as researchers as well as to children's peers) appeared to support candid reflection, including our use of prompts to explore alternative perspectives and approaches such as writing on post‐it notes independently to then discuss together, yet perceived expectations and group formats may still have shaped responses. Being a case study school may have introduced pressure to demonstrate success, raising questions about transferability. Data were collected during or shortly after delivery, meaning initial ‘impact’ reflections often focused on engagement; follow‐ups on longer‐term impacts were not possible due to transition to new teachers.

## CONCLUSIONS

The current study offers insights into the complex realities of implementing SEL interventions like Passport in English schools. By examining the perspectives and experiences of children, teachers and senior leaders, we highlight how programme features such as rich narrative approaches, structured reflection space and inclusion of carefully guided sensitive content on grief and loss are understood to shape engagement, coping flexibility and expanded emotional literacy. Findings foreground children's agency in engaging with and making sense of the strategies included in initiatives targeting their social and emotional development. The study also highlights that teachers' interpretations play a central role in how interventions are enacted in practice, and that pedagogical value, practitioner endorsement, and perceived possibilities for impact can diverge in saturated SEL contexts. That illustrates how differentiation failure may coexist with meaningful learning and pedagogical experiences. Collectively, the study advances educational psychology theory by demonstrating how fidelity, adaptation, contextual fit, programme format, and approaches intersect in practice to support or constrain learning—including for some groups more than others—and underscores the importance of multi‐perspectival qualitative inquiry in illuminating the nuanced mechanisms underlying school‐based SEL interventions.

## AUTHOR CONTRIBUTIONS

**Ola Demkowicz:** Conceptualization; formal analysis; funding acquisition; methodology; project administration; supervision; validation; visualization; writing – original draft. **Annie O'Brien:** Methodology; data curation; investigation; formal analysis; writing – original draft; writing – review and editing; project administration. **Suzanne Hamilton:** Methodology; data curation; investigation; formal analysis; project administration; writing – original draft; writing – review and editing. **Lauren Burke:** Methodology; data curation; investigation; formal analysis; project administration; writing – review and editing. **Melek Alemdar:** Methodology; data curation; investigation; formal analysis; writing – original draft; writing – review and editing; project administration. **Carla Mason:** Formal analysis; writing – review and editing; data curation; methodology. **Molly Anderton:** Methodology; writing – review and editing; formal analysis. **Pratyasha Nanda:** Methodology; formal analysis; writing – review and editing. **Lily Corke Butters:** Methodology; formal analysis; writing – review and editing. **Felicitas Beissel:** Writing – review and editing. **Yizhuo Lu:** Methodology; data curation; investigation; formal analysis; project administration; writing – review and editing. **Eleanor Chatburn:** Validation; writing – review and editing. **Neil Humphrey:** Conceptualization; methodology; validation; supervision; funding acquisition; project administration; writing – review and editing. **Pamela Qualter:** Conceptualization; methodology; validation; supervision; funding acquisition; writing – review and editing; project administration.

## FUNDING INFORMATION

This research is supported by the Kavli Trust under the award Kavli‐2021‐0000000019. MAl is funded by The Scientific and Technological Research Council of Turkey (TÜBİTAK) 2219 Program.

## CONFLICT OF INTEREST STATEMENT

The authors declare no conflicts of interest.

## Supporting information


Table S1.

Appendix S1.

## Data Availability

Data will be made openly available at the end of the trial.

## Footnotes

^1^
Passport for Life was originally developed and implemented in Canada and has previously only been evaluated in a small‐scale Canadian trial.
