# Supplementary material for: Child and school staff perceptions and experiences of universal social and emotional learning curricula in context: A qualitative case study registered report examining ‘Passport Skills for Life’
Source: Br J Educ Psychol. 2026 Mar 2;96(3):1055–79. doi: 10.1111/bjep.70065 (PMC13440978; doi:10.1111/bjep.70065)
Supplement: Supplementary file 1 [file BJEP-96-1055-s001.docx]

**Supplementary file**

| Table S1  Codes underpinning subthemes and major codes | | |
| --- | --- | --- |
| Theme/code | Sources | References |
| 1. Implementation processes | 28 | 235 |
| a. Dosage | 12 | 16 |
| All Passport lessons were delivered | 6 | 7 |
| One class did not do celebration | 1 | 1 |
| Lessons were not always delivered weekly | 6 | 7 |
| Delivered across 3 week period due to competing programmes | 1 | 3 |
| Sometimes delivered more than 1 lesson to catch up | 2 | 2 |
| Specific slot in timetable for weekly delivery | 5 | 6 |
| b. Adaptations or modifications | 10 | 59 |
| Adapted to mimic or align with other programmes | 1 | 1 |
| Changes made or planned in response to perceived or actual pupil need or engagement | 7 | 30 |
| As teacher becomes more familiar, makes more structural changes to suit class | 1 | 1 |
| Changed activities to deliver content more appropriately for my class | 1 | 1 |
| Changed to individualised or paired tasks e.g. for engagement, keep it interesting | 3 | 5 |
| Changes in response to student engagement | 3 | 6 |
| I changed something based on how I think my class would react | 4 | 5 |
| I would adapt, restructure, or drop Passport based on my student's needs at that time | 3 | 4 |
| Need to listen and adapt to class needs to do it well | 5 | 8 |
| Did not go well | 1 | 1 |
| Easy to make changes and adapt on the go | 3 | 3 |
| I skimmed or combined lessons to complete programme | 1 | 2 |
| Lessons adapted or augmented to produce written work | 7 | 23 |
| Activities adapted to produce personal piece of work (for Ofsted, for child to refer back to, for 'proof' of work done) | 6 | 15 |
| Adding individualised tasks when lessons ran short | 1 | 1 |
| Written work to embed learning, children can return to, something personal for them | 3 | 4 |
| b. Fidelity | 16 | 32 |
| Activity timings were a guide, not a rule | 11 | 16 |
| Adaptations... There were often changes made to activity timings | 8 | 9 |
| Cut straight to the point rather than include cusioning Passport provides | 2 | 2 |
| Discussion time often reduced | 1 | 1 |
| Game omited or shortened | 3 | 3 |
| Positive feedback on the lesson timings | 1 | 1 |
| Time for indepth and personalised discussion | 1 | 1 |
| Shorter lesson fits school schedule | 1 | 1 |
| Split into two sessions to cover all content (teacher quality) | 1 | 1 |
| Lessons are under recommended time | 3 | 3 |
| Lessons are usually one hour | 3 | 3 |
| Lessons were merged due to similar learning outcomes | 1 | 1 |
| Classifying and prioritising activities based on their perceived importance | 1 | 1 |
| Games were not played | 6 | 11 |
| Children were not interested | 1 | 1 |
| Due to time | 3 | 4 |
| Game not played because perceived as too young for class | 1 | 1 |
| Games were omited because they were unique to Passport and perceived as supplement rather than key for for learning outcomes | 2 | 2 |
| No reason - perhaps perceived as supplementary rather than core component to learning outcomes | 3 | 3 |
| Introduction or conclusion were often omitted | 4 | 15 |
| I selected and delivered 'best bits' to navigate competing demands | 1 | 1 |
| Introduction or conclusion were often omitted | 3 | 11 |
| Parent worksheets not used due to school policy | 1 | 1 |
| Sticking to how it should be delivered | 2 | 3 |
| Prescription to, and prioritising of, the main activity | 1 | 2 |
| Use of lesson materials varied across time and between classrooms | 7 | 14 |
| Classroom resources used once | 1 | 1 |
| Comics usually delivered as animation | 2 | 2 |
| Resources used for reference around classroom sparingly | 3 | 3 |
| The Passports were typically used once at the start but not throughout | 6 | 8 |
| c. Quality | 9 | 14 |
| Additional time spent planning and personalising Passport for my class | 3 | 3 |
| Creating additional resources in own time | 1 | 1 |
| Some teachers don't have time for this | 1 | 1 |
| Our teacher seems happy during Passport | 2 | 5 |
| I enjoy this opportunity to chat about what's meaningful for them | 1 | 1 |
| Personalised and individualised approach to lesson delivery | 2 | 3 |
| Lessons are made more accessible to reach lower ability children e.g., more time, mixed ability pairing | 2 | 3 |
| I would provide additional individualised sessions for those who need it | 1 | 1 |
| Using knowledge of the students to deliver content mindfully and appropriately e.g., trigger warning for death | 3 | 3 |
| d. Reach and responsiveness | 17 | 40 |
| All children have a voice and a say during Passport lessons | 6 | 9 |
| Willingness to share during lessons | 1 | 1 |
| Apply what we're learning outside of Passport lessons | 1 | 1 |
| Children are relaxed and engaged compared to traditional lessons | 4 | 7 |
| Children enjoyed the situations, scenarios and role plays | 3 | 3 |
| Enjoy the spoken vs written approach to problem solving and learning | 2 | 3 |
| Visuals and activities make it accessible to subgroups | 1 | 2 |
| Children understand the importance of the topics they're engaging with | 1 | 1 |
| Enthusiasm, excitement and enjoyment around Passport lessons | 7 | 12 |
| Age appropriatness of activities and mystical theme | 6 | 8 |
| Children enjoy the engaging materials | 5 | 18 |
| Materials that can be personalised by them | 7 | 10 |
| They loved the game | 1 | 1 |
| Lesson format and structure are engaging | 2 | 3 |
| Loved and cared for the characters | 4 | 5 |
| They love that Passport lessons, and passport booklets, are for them | 2 | 3 |
| Some children get upset during lessons | 3 | 3 |
| The relatability and seriousness of topic is hindered by the mythical theme | 2 | 4 |
| Topics are relatable and relevant to the children | 2 | 2 |
| e. Programme differentiation | 20 | 74 |
| Broadly similar content to wider programmes and PHSE resource | 3 | 3 |
| Teacher accidentally talking about other programme as Passport | 1 | 1 |
| Very similar to others in terms of topics but passport feels easier to deliver not as much burden on teacher | 2 | 2 |
| Distinctness of approach and engaging nature | 3 | 3 |
| Positively different for other schemes as it has open discussion which is nice for children | 1 | 1 |
| Very similar to others in terms of topics but passport feels easier to deliver not as much burden on teacher | 2 | 2 |
| Passport is complementary component of wider PHSE and whole school provision | 17 | 49 |
| Other programmes needed to top up what passport does not cover like citizenship | 1 | 1 |
| Other SEL programs used at the school | 9 | 18 |
| Other types of training or lessons which can address SEL which is not passport | 3 | 3 |
| Passport replace or complements PSHE | 6 | 16 |
| Providing targeted support for emotional needs | 1 | 1 |
| Push for well being in schools training generally around how to respond to childens emotions | 1 | 1 |
| Supporting MH and SEL skill development is a school priority | 2 | 5 |
| Creating experiences and opportunities for disadvanted pupils | 1 | 1 |
| School provided internal training in MH or SEL | 2 | 2 |
| Targeted inteventions for students | 3 | 4 |
| Passport is different from other programmes or training | 7 | 12 |
| Some content differentiation; broad range of relevant topics as positive | 3 | 5 |
| 2. Factors affecting implementation | 25 | 198 |
| a. Programme characteristics | 21 | 104 |
| Engagement and friendliness | 10 | 21 |
| A child friendly approach that helps children access serious themes | 3 | 3 |
| Children friendly activties made it easier to implement as children were more engaged | 1 | 1 |
| Children friendly activties made it easier to implement as children were more engaged (2) | 1 | 1 |
| Children see it as different to traditional lessons | 3 | 8 |
| Correct order of topics easing childen into potentially more difficult topics | 2 | 2 |
| Happy to finish as does not enjoy passport when the whole lesson is talking | 1 | 1 |
| How passport does not feel like real school work good but can cause behaviour issues | 1 | 1 |
| Need for activities to be less of the same to stop children getting bored | 1 | 1 |
| Need for activities to be used to keep engagement | 1 | 1 |
| Passport is interactive which is good for children | 1 | 1 |
| The flow and build of passport makes it easier to deliver and for pupils to understand | 1 | 1 |
| Matching the needs of the classroom | 8 | 13 |
| Need to be aware of individual needs and response to certain topics and differences across class | 2 | 2 |
| Need to be considerate of your class, religion meant some topics need to be taught more carefully | 1 | 1 |
| Sessions wellsuited to the classroom's needs | 5 | 9 |
| Use of passport is really dependent on the children in your school and their needs | 1 | 1 |
| Optimising program delivery and resource use | 15 | 55 |
| 'Optional' activities to use when you have done everything would be good | 1 | 1 |
| Clarification needed on use of resources for teachers | 1 | 1 |
| Criticism for game | 3 | 6 |
| critisizm for the comic strip&videos | 3 | 7 |
| Comic strip&videos are short | 4 | 13 |
| critisizm for the teacher's pack | 4 | 6 |
| Critism for the boxes | 1 | 1 |
| Difficult to navigate exactly how to deliver passport | 1 | 1 |
| Further clarity of how to adapt needed | 1 | 1 |
| Implementation made easier when teacher is able to know structure and what is coming next | 1 | 1 |
| Manual is clear and self explanatory | 2 | 2 |
| Materials make it easier to continue | 1 | 1 |
| Missing more detail around transition (school and classes) | 1 | 1 |
| Need for a variety of activities and more examples in resources of what to add in as part of sessions when extra is needed | 2 | 2 |
| Need for further explanations of activities might help teachers make them last the full session | 1 | 1 |
| Need for further focus on meaning of emotions not just situations | 1 | 1 |
| Need for setting rules and boundaries as discussing emotions | 1 | 1 |
| Need to push inclusion of activities that allow children to express themselves | 3 | 3 |
| Needs to be less explaining from the teacher and talking | 1 | 1 |
| Passport masterfolder is self explanatory | 1 | 4 |
| Resources can be confusing to work out | 1 | 1 |
| Some emotions were not familiar to children interpreted differently by children than what passport meant | 1 | 1 |
| Sometimes group discussions do not respect privacy for children | 2 | 2 |
| Suggest more independent work when topics are sensitive, to provide opportunity to be kept private | 2 | 2 |
| The flow and build of passport makes it easier to deliver and for pupils to understand | 1 | 1 |
| Too much material need to be condensed | 1 | 2 |
| Unmotivated or bored by Passport | 2 | 4 |
| Design is childish, unengaging, boring | 2 | 3 |
| It's repetitive | 1 | 1 |
| It's too slow; they lose interest e.g., the comic animation | 1 | 2 |
| Timing trade offs Navigating too much time and too little | 8 | 13 |
| Adaptable timings make it easy to schedule and fit around school timetable | 2 | 3 |
| Children reporting how there is no time to finish work and more than one topic covered in an hour | 1 | 1 |
| Lessons are too long to keep attention | 1 | 1 |
| More variety of suggested activities as it is hard to fill the time | 1 | 1 |
| Need to consider time of day as can be too much first thing in a morning | 1 | 1 |
| Not enough in session layout to make it cover the hour | 1 | 1 |
| Not enough time to deliver whole lesson | 1 | 1 |
| Programme length is perfect | 1 | 2 |
| Too much material need to be condensed | 1 | 2 |
| b. School factors | 13 | 53 |
| Alignment with current practices and needs | 11 | 20 |
| Aligned with school priorities eg MH, SEL | 9 | 11 |
| School mission-policy alignment | 2 | 4 |
| How passport fits into the current school timetable | 5 | 9 |
| Easily fits into schedule as it can replace PSHE | 1 | 1 |
| Finding time in a busy curriculum | 2 | 5 |
| Increases workload - new scheme | 2 | 3 |
| Impact of Student and Family Background on Implementation | 10 | 32 |
| Background of the students | 7 | 23 |
| Classroom characteristics | 4 | 11 |
| Need for passport and SEL development due to area school is in and the background or family set up | 2 | 2 |
| School culture | 6 | 10 |
| School climate | 6 | 9 |
| School Environment and Leadership Impact on Implementation | 1 | 1 |
| Availability of SLT to discuss SEL needs of childen is important | 1 | 1 |
| Positive teacher relationships creating a positive school environment | 2 | 4 |
| School environment fosters positive student behaviours | 2 | 4 |
| Positive teacher relationships creating a positive school environment | 2 | 4 |
| School environment fosters positive student behaviours | 2 | 4 |
| Space in school impacts what can be done activity wise | 1 | 1 |
| Teachers given autonomy with timetable | 4 | 5 |
| Supportive staff and SLT | 6 | 11 |
| The physical structure of the school | 1 | 1 |
| c. Teacher factors | 14 | 26 |
| Perceived Delivery and Resource Navigation | 11 | 17 |
| Teacher didnt like delievering some of passport as it stands but see promise | 1 | 3 |
| Teacher enjoyed teaching Passport | 1 | 1 |
| Teacher needs more autonomy on the structure and content | 1 | 1 |
| Teacher's classroom ethos | 4 | 6 |
| Years of experience teaching | 6 | 6 |
| Teacher buy-in and commitment to passport intervention and pupils | 5 | 9 |
| Importance of teacher buy-in to embed the approach | 1 | 1 |
| Our teacher is kind and responsive to our emotions | 1 | 1 |
| Our teacher seems bored because we know this stuff | 1 | 1 |
| Positive teacher attitudes towards SEL and or Passport | 2 | 6 |
| External factors | 8 | 15 |
| Teacher Training Perceptions and Recommendations | 8 | 15 |
| May help if there is communication with other schools or teachers delivering it | 1 | 1 |
| Need for more suppport training and to potentially tailor training to learning preference of teacher (more visual or less examples) | 3 | 3 |
| Need for other training elements more real life examples | 2 | 2 |
| Online information has been good and easily accessible | 1 | 1 |
| Potential school visits to see passport being delievered in other places to improve training | 1 | 1 |
| Recommendations for the training | 2 | 2 |
| Teacher did not attend booster session | 5 | 5 |
| 3. Perceptions of impact | 23 | 284 |
| a. Perceived universal impact | 23 | 176 |
| Bullying - helps children manage or cope with bullying | 4 | 4 |
| Helped children develop more coping strategies for different situations | 14 | 39 |
| Children apply Passport learning outside of sessions | 2 | 2 |
| Has helped children cope with academic challenges | 2 | 4 |
| Has increased repertoire of coping strategies | 6 | 9 |
| Helped children cope with change | 5 | 5 |
| Helped to prepare for next year or high school or future situations | 3 | 5 |
| Helps you cope with stress if its done frequently | 1 | 1 |
| Learnt how to cope with different scenarios in life | 9 | 13 |
| Learnt that there are multiple solutions to problems | 3 | 4 |
| Helped children understand, express and regulate emotions | 16 | 82 |
| Better understanding and reporting of their own emotions | 2 | 2 |
| Has helped children with feelings about themselves | 7 | 11 |
| Increase in confidence and self-belief | 5 | 7 |
| Increased self-awareness and self-acceptance | 3 | 4 |
| Helped children cope with feelings of grief or loss | 5 | 9 |
| Improved emotional literacy | 8 | 13 |
| Increased understanding of others' emotions | 6 | 9 |
| Learnt how to better manage or control emotions | 8 | 29 |
| Benefits in particular with dealing with frustration | 1 | 1 |
| Since Passport everything has calmed down a lot with each other | 1 | 1 |
| More able to talk about feelings with adults and others | 6 | 9 |
| Improved children's social and relationship skills | 11 | 33 |
| Has helped children with general interaction and relationship skills | 4 | 6 |
| Helped children manage or reduce conflict or arguments in relationships | 6 | 13 |
| Helped with friendships - making friends and being friends | 3 | 8 |
| Reminds children to be kind to others | 1 | 4 |
| Teaches us to respect that everyone is unique and to be yourself | 2 | 2 |
| Improved relationship with teacher | 1 | 1 |
| Increased help-seeking | 4 | 5 |
| Learnt who talk to when you feel lonely | 1 | 1 |
| Learnt that others have similar experiences to their own | 1 | 1 |
| Taught children they can speak to someone about their emotions | 4 | 4 |
| Ways in which Passport has been less helpful or not helped | 6 | 10 |
| Children sometimes struggle to apply learning or make link to behaviour to benefit from Passport | 3 | 3 |
| No improvements in behaviour | 1 | 1 |
| Passport might not help in the long term or for more complex situations | 2 | 3 |
| Some strategies are not helpful | 1 | 1 |
| Some strategies make me sad or more upset | 2 | 2 |
| b. Perceived impact among specific groups | 15 | 88 |
| Benefits for children who have intersecting characteristics | 2 | 5 |
| Benefits for teachers | 3 | 4 |
| Creates confidence in teachers | 2 | 2 |
| Helps teacher keep calm | 1 | 1 |
| Helps teacher-pupil relationship | 1 | 1 |
| Boys and girls have benefitted differently | 5 | 12 |
| Boys less responsive than girls because of relevancy | 1 | 1 |
| Boys struggling with anger lack self-awareness to learn from lessons on anger at first but this does sink in | 1 | 2 |
| Girls have benefitted less (than boys) because they are more mature and don't have as many friendship problems | 1 | 2 |
| Has given girls the opportunity to talk about family relationships in school | 1 | 1 |
| Has helped boys with friendship problems and falling out over football - more need among boys | 1 | 2 |
| Has helped girls talk about anxiety and process emotions | 1 | 2 |
| Has helped girls with friendships and falling outs | 1 | 1 |
| No difference between boys and girls; its just individual cases | 1 | 1 |
| Differential benefit for socio-economically disadvantaged children | 6 | 20 |
| 'Privileged' children have fewer difficulties but benefit by learning about others | 1 | 1 |
| Children from disadvantaged families have benefitted more through learning more coping strategies | 2 | 2 |
| Children with difficult home lives have benefitted more than others | 4 | 12 |
| Children with more secure upbringing benefit less at present | 3 | 5 |
| Lower achieving pupils more able to identify and express emotions since Passport | 2 | 4 |
| Mixed benefit for children with SEND | 5 | 12 |
| Benefits for children with ADHD | 2 | 3 |
| Children with SEND are left out because there is nothing for them to do | 1 | 2 |
| Helped children with SEND to identify, express, and respond to feelings | 1 | 2 |
| Passport needed to be differentiated to meet individual needs of children with SEND and EHCPs | 2 | 3 |
| The benefit for SEND children can be dependent on the mood of the child on that day | 1 | 2 |
| Particular benefit for EAL children through emotion vocab and literacy | 3 | 3 |
| Particularly beneficial for children with social or emotional difficulties | 7 | 18 |
| Helpful for children who struggle with anger or have a 'temper' | 4 | 9 |
| Less benefit for children who are mature or who can already process emotions | 3 | 4 |
| Particular benefit for children with low self-esteem | 1 | 3 |
| Passport is a good tool for helping children with SEMH needs but more intensive intervention needed | 1 | 2 |
| How do teachers measure impact - what does impact look like for teachers | 7 | 13 |
| Benefit depends on specific needs of class overall | 1 | 1 |
| Changes in children could just be because they have matured naturally | 1 | 1 |
| Children benefit from Passport just like other lessons because it's being taught to them directly | 1 | 1 |
| Children can tell teacher how to cope with something | 1 | 1 |
| Delayed impact - changes have come later in the year | 1 | 1 |
| Difficult to directly see immediate benefit after only 5 or 6 lessons | 1 | 3 |
| Everybody understands it and benefits just from knowing | 1 | 1 |
| Teachers talk about engagement or enjoyment when asked about benefit | 3 | 4 |
| Mechanisms and factors that help or hinder | 5 | 6 |
| Ability to apply learning to real life | 1 | 2 |
| Influence of perceived academic achievement | 1 | 1 |
| It has worked because it is relevant for children's needs at this age | 2 | 2 |
| Supportive environments at home - socialisation practices at home influence impact of Passport | 1 | 1 |
| 4. Sustainability | 6 | 19 |
| a. Openness to continuation of programme as part of a wider set of PHSE practices | 3 | 6 |
| b. Teachers planning to continue using specific resources and ways of engaging with children in day-to-day practice | 5 | 7 |
| c. Factors affecting sustainability | 3 | 6 |
