# Supplementary material for: Child and school staff perceptions and experiences of universal social and emotional learning curricula in context: A qualitative case study registered report examining ‘Passport Skills for Life’
Source: Br J Educ Psychol. 2026 Mar 2;96(3):1055–79. doi: 10.1111/bjep.70065 (PMC13440978; doi:10.1111/bjep.70065)
Supplement: Supplementary file 2 [file BJEP-96-1055-s002.docx]

**Supplementary Materials**

Table S1

*The Content of the Passport Modules and Sessions*

| **Module** | **Topic of the session** | **Goal of the session** |
| --- | --- | --- |
| Emotions | 0. Beginning the adventure: the meeting | To stimulate children’s interest in participating in Passport programme activities. |
|  | 1. Valuing our differences and similarities | To help children identify, appreciate and value their differences and similarities. |
|  | 2. Understanding and expressing our emotions | To help children identify, name and talk about their own emotions, and to understand that we can sometimes feel different emotions at the same time. |
|  | 3. Recognising other people's feelings | To identify and then communicate our recognition of the emotions that someone else is feeling and to acknowledge that a person can feel more than one emotion at the same time, and that these emotions can sometimes be contradictory |
| Relationships and helping each other | 1. Helping each other and coping skills | To identify and evaluate coping skills which are focused on actions as well as those which are focused on emotions and to learn how to offer, ask for and accept help. |
|  | 2. Friendship | To explore how to make and keep friends. |
|  | 3. Challenges in friendship | To understand our reactions when we have to deal with rejection, abandonment and disappointment, and to identify coping strategies for dealing with these situations. |
| Difficult situations | 1. Dealing with frustration | To identify our reactions and coping skills in frustrating situations or in situations where we do not get our own way. |
|  | 2. Dealing with stress | To identify stress and how it feels inside, and to identify coping skills for dealing with it. |
|  | 3. Dealing with conflict | To identify and practise different coping skills in situations that involve conflict. |
| Fairness, justice and what is right | 1.Unfairness in daily life | To identify our feelings in unfair and unjust situations and to recognise our coping skills in these situations and to learn new ones. |
|  | 2. Dealing with bullying | To identify stress and how it feels inside, and to identify coping skills for dealing with it. |
|  | 3. Unfairness and injustice in the world | To identify our emotions when dealing with injustice and to find coping skills for dealing with these situations. |
| Change and loss | 1. Coping with change | To explore the consequences of change, our reactions to change, and to learn coping strategies for dealing with it. |
|  | 2. Coping with loss | To identify stress and how it feels inside, and to identify coping skills for dealing with it. |
|  | 3. Helping others in difficult situations | To identify our emotions when dealing with injustice and to find coping skills for dealing with these situations. |
|  | 4. Let's review – coping kit | To review everything we have learned in Passport. |
|  | 5. Celebration! | To celebrate what we have learned in Passport. |

Table S2

*Implementation dimensions*

| **Dimension** | **Definition** |
| --- | --- |
| Fidelity | Degree to which the programme is delivered as intended by the developer |
| Dosage | The number of programme sessions delivered |
| Quality | How well a programme is delivered by the programme facilitator |
| Reach | The proportion of participants in attendance during programme delivery |
| Responsiveness | Participant engagement with the programme |
| Programme differentiation | The extent to which the programme is distinct from existing practices |
| Monitoring of control groups | Monitoring and understanding the untreated comparison conditions |
| Adaptations | Changes made to the programme during delivery |
